# Physiological Predictors of Maximal Incremental Running Performance

**DOI:** 10.3389/fphys.2020.00979

**Published:** 2020-08-05

**Authors:** Fábio J. Lanferdini, Edson S. Silva, Esthevan Machado, Gabriela Fischer, Leonardo A. Peyré-Tartaruga

**Affiliations:** ^1^Laboratório de Biomecânica, Centro de Desportos, Universidade Federal de Santa Catarina, Florianópolis, Brazil; ^2^Laboratório de Pesquisa do Exercício, Escola de Educação Física, Fisioterapia e Dança, Universidade Federal do Rio Grande do Sul, Porto Alegre, Brazil

**Keywords:** runners, incremental test, VO_2MAX_, running economy, metabolic cost

## Abstract

**Purpose:**

The aim of this study was to verify whether physiological components [vertical jumps (Squat Jump – SJ and Countermovement Jump – CMJ), eccentric utilization ratio (EUR) of vertical jumps, running economy (RE), metabolic cost (C_*MET*_), first and second ventilatory threshold (VT_1_ and VT_2_) maximal oxygen uptake (VO_2MAX_)] can predict maximal endurance running performance.

**Methods:**

Twenty male runners performed maximal vertical jumps, submaximal running at constant speeds, and maximal incremental running test. Before, we measured anthropometric parameters (body mass and height) and registered the training history and volume. SJ and CMJ tests were evaluated prior to running tests. Initially, the oxygen uptake (VO_2_) was collected at rest in the orthostatic position for 6 min. Soon after, a 10-min warm-up was performed on the treadmill at 10 km⋅h^–1^, followed by two 5-min treadmill rectangular tests at 12 and 16 km⋅h^–1^ monitored by a gas analyzer. After that, the runners performed a maximal incremental test, where the VT_1_, VT_2_, and VO_2MAX_ were evaluated, as well as the maximum running speed (vVO_2MAX_). Thus, RE and C_*MET*_ were calculated with data obtained during rectangular running tests. Multivariate stepwise regression analyses were conducted to measure the relationship between independent variables (height and power of SJ and CMJ, EUR; RE and C_*MET*_ 12 and 16 km⋅h^–1^_;_ VT_1_, VT_2_, and VO_2MAX_), as predictors of maximal running performance (vVO_2MAX_), with significance level at α = 0.05.

**Results:**

We found that VO_2MAX_ and RE at 16 km⋅h^–1^ predict 81% of performance (vVO_2MAX_) of endurance runners (*p* < 0.001).

**Conclusion:**

The main predictors of the maximal incremental running test performance were VO_2MAX_ and RE.

## Introduction

Improvements of endurance running performance are based on improvements of the physiological predictors such as the maximal oxygen uptake (VO_2MAX_), running economy (RE) and metabolic thresholds (McLaughlin et al., 2010). The determinants of the endurance performance model demonstrate how the individual’s VO_2MAX_ determines the upper limit of aerobic metabolism (Bassett and Howley, 2000). Therefore, VO_2MAX_ has an important relationship with endurance running performance (McLaughlin et al., 2010). However, trained runners may have similar VO_2MAX_ values and thus other physiological indexes can contribute for the success of predominantly aerobic events such as RE and lactate threshold (Kipp et al., 2019).

Running speed is then determined by how efficiently the corresponding oxidative adenosine triphosphate turnover at the fractional utilization of VO_2MAX_ is converted to locomotion (i.e., RE) (Joyner and Coyle, 2008). Metabolic effectiveness refers to the utilization of available energy to provide optimal performance, whereas cardiopulmonary efficiency to least work output for the processes related to oxygen transport and utilization (Daniels, 1985; Saunders et al., 2004; Peyre-Tartaruga and Coertjens, 2018). Therefore, RE is an important physiological determinant for the endurance performance (Daniels, 1985; Kipp et al., 2019). Improvements in RE allow athletes to run at a faster velocity for the same oxygen uptake (VO_2_) and thus achieve superior performances (Hoogkamer et al., 2016, 2017). Accordingly, ∼1% improvements in RE should directly translate to ∼1% faster running 3000 m of running (Hoogkamer et al., 2016).

Another approach used to evaluate the metabolic economy in distance running is the metabolic cost (C_*MET*_) for running, which is independent of the speed during indoor tests (Arellano and Kram, 2014). Therefore, the amount of metabolic energy used to run a given distance is the same (Margaria et al., 1963; Arellano and Kram, 2014; Lacour and Bourdin, 2015). In addition, according to di Prampero et al. (1986), runners with higher VO_2MAX_ (direct relation) and lower C_*MET*_ (inverse relation), could present better performance. However, no study has been found to examine the relationship between C_*MET*_ and the velocity at VO_2MAX_ (vVO_2MAX_).

Furthermore, muscle strength or power are other important factor in predicting endurance running performance (Dumke et al., 2010), which can be assessed by performing maximal vertical jumps [squat jump (SJ) and countermovement jump (CMJ), more similar to the motor gesture performed during the race]. Previous research showed that improved strength and power results in better RE and performance (Balsalobre-Fernandez et al., 2016). This may occur probably by the changes in muscle power and tendon stiffness (Kubo et al., 2007). Therefore, the performance of vertical jumps (height and power), could be related to the maximum performance of runners.

Accordingly, relating all these physiological aspects to the maximum running performance can help coaches and runners understand which features can predict performance and decide whether it is important to offer time for training specific system adaptation during regular running training. Our goal was to verify the relationship between physiological parameters [VO_2MAX_, first and second ventilatory threshold (VT_1_ and VT_2_), RE, C_*MET*_ and height and power of vertical jumps (SJ and CMJ) and eccentric utilization ratio (EUR) of vertical jumps] on prediction of maximal incremental running performance (maximum running speed - vVO_2MAX_). We hypothesized that the determinants of maximal incremental running performance were VO_2MAX_ and lower RE.

## Materials and Methods

### Participants

Twenty male recreational runners, with ∼34 ± 8 years of age, participated in this study. Running experience was ∼5.5 years, and training volume was ∼63 ± 32 km/week. Before the selection interview, all procedures were presented to the participants, who signed a consent form to participate in the study that was approved by the local ethics committee (No. 2.437.616), according to the Declaration of Helsinki. Male runners with ages between 18 and 45 years, 2 years of running experience, training volume of at least 30 km/week and reaching a minimum speed of 19 km⋅h^–1^ in the incremental test were included in the present study. Participants were excluded if (1) they had any musculoskeletal injury of the lower and/or the upper limbs; (2) they had any contra-indications for maximal effort (cardiovascular, musculoskeletal, respiratory, or neurologic diseases); and (3) they had any difficulty in understanding and/or executing of the tests.

### Experimental Design

**FIGURE 1:**
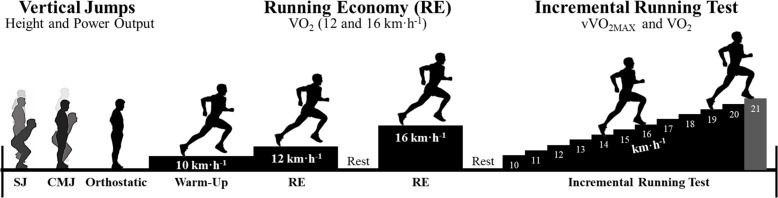
Experimental design.

Runners attended to one testing day in the laboratory. Anthropometric, maximal vertical jumps (SJ and CMJ), constant running speed, and maximal incremental running test [VO_2_ was measured] were performed (Figure 1).

### Procedures

#### Anthropometry

Anthropometry was evaluated according to the International Society for Advancement of Kineanthropometry (Marfell-Jones et al., 2012).

#### Vertical Jumps (SJ and CMJ)

The runners performed the SJ and CMJ using a jumping mat (Jump System Pro^®^, CEFISE, Nova Odessa, Brazil), with sample rate of 1000 Hz. Each athlete performed three attempts of the SJ and CMJ with maximal effort. Thirty seconds between attempts were given to each runner. Both vertical jumps were performed according to the recommendations of Bosco et al. (1983). The runners were instructed to jump as high as possible, with their hands on their hips. The variables used in the data analysis were jump height (cm), power output (PO) and PO normalized by the body mass (W⋅kg^–1^). In addition, the eccentric utilization ratio (EUR) was calculated: EUR jump height [EUR-HJ (CMJ-SJ); (ΔCMJ-SJ); and (CMJ/SJ)] and EUR of peak power output [EUR-PO (CMJ-SJ); (ΔCMJ-SJ); (CMJ/SJ)]. Pre-stretch augmentation can be calculated as a percentage with percentage pre-stretch increase [EUR (ΔCMJ-SJ)]. Additional approach is to amount reactive strength [EUR (CMJ-SJ)], as described in McGuigan et al. (2006).

#### Running Economy and Metabolic Cost

All the participants underwent familiarization on a treadmill (Super ATL, Inbrasport-Inbramed, Porto Alegre, Brazil). The VO_2_ was measured breath by breath during the incremental test using an open-circuit indirect calorimetry system (Cosmed, Quark CPET, Rome, Italy). Initially, the VO_2_ was collected at rest in the orthostatic position for 6 min. Soon after, a 10-min warm-up was performed on the treadmill at 10 km⋅h^–1^, after which the constant speed running test was performed for 5 min at 12 and 16 km⋅h^–1^, with a 5-min interval between each test (Saunders et al., 2004). The treadmill velocity was calibrated before tests (Mocap System), by digitizing an adhesive retro-reflective marker on the tread belt as it traveled along the length of the treadmill. In addition, parameters including room temperature and humidity were measured using the same gas analyzer (Cosmed, Quark CPET, Rome, Italy). The VO_2_ curves from RE tests were analyzed using the software PFT ergo (Cosmed, Quark CPET, Rome, Italy), and the mean VO_2_ values were calculated and plotted at the last minute of each bout. RE was defined as the relationship between VO_2_ and the running velocity (Daniels, 1985). The metabolic power (W_*MET*_) was considered the difference between the VO_2_ measured during exercise and the VO_2_ at rest. Because the unit of measure used was Watts (W), this difference was multiplied by the energy coefficient (20.9 J⋅mL^–1^) and divided by the time in seconds (60 s). The C_*MET*_ values relative to the speeds of 12 and 16 km⋅h^–1^ were calculated by dividing W_*MET*_ by the speed in m⋅s^–1^ (di Prampero et al., 1986).

#### Maximum Incremental Test

The runners were submitted to a maximal incremental test (Figure 1). It started with an initial velocity of 10 km⋅h^–1^, and 1 km⋅h^–1^ was added per minute until exhaustion (Bentley et al., 2007). The VO_2_ obtained during the maximal incremental test was evaluated on the treadmill and followed a similar gas analysis as described above.

The VO_2_ analysis during the maximal incremental test was performed by visual inspection. VO_2_ values were plotted to exclude values with four standard deviations above or below the average of the movable windows of the whole curve—average of three breaths in each window (Fernandes et al., 2012). During the maximum incremental test, the vVO_2MAX_ was obtained from the last completed stage, while the VO_2MAX_ was determined as the highest value observed in the last test stage (Bentley et al., 2007). Also, gas exchange data were analyzed to define the VT_1_ and VT_2_, as described in Bentley et al. (2007).

### Statistical Analysis

Data normality and homogeneity were assessed by the Shapiro-Wilk and Mauchly tests, respectively. Stepwise multiple linear regression method was used to estimate the relative contributions of independent variables [SJ (cm); PO-SJ (W); PO-SJr (W⋅kg^–1^); CMJ (cm); PO-CMJ (W); PO-CMJr (W⋅kg^–1^); EUR-HJ (CMJ-SJ); EUR-HJ (ΔCMJ-SJ); EUR-HJ (CMJ/SJ); EUR-PO (CMJ-SJ); EUR-PO (ΔCMJ-SJ); EUR-PO (CMJ/SJ); VO_2MAX_ (ml⋅kg^–1^⋅min^–1^); VT_2_ and VT_1_ (ml⋅kg^–1^⋅min^–1^); RE 12 and 16 km⋅h^–1^ (ml⋅kg^–1^⋅min^–1^); C_*MET*_ 12 and 16 km⋅h^–1^ (J⋅kg^–1^⋅m^–1^)] on the dependent variable of the performance [vVO_2MAX_ (km⋅h^–1^)]. Our collinearity diagnostic exploration resulted in variance inflation factors of <2.0 and tolerance of 0.10–0.70, which indicate acceptable levels of multicollinearity of the variables (Dormann et al., 2012). Statistical analysis was performed with SPSS 20.0 for Windows, with a significance level of α = 0.05.

## Results

Anthropometric, physiological, training characteristics and performance of runners are summarized in Table 1.

**TABLE 1:** Descriptive values of all variables evaluated of runners.

	**Mean**	**SD**	**CV (%)**	**Range**
Age (years)	34.0	7.9	23.4	22–48
Body mass (kg)	67.2	6.1	9.1	55.6–80.2
Height (cm)	173.6	7.0	4.1	160–186
Experience (years)	5.5	2.5	45.1	3–14
Training frequency (days/week)	7.6	6.8	89.1	25–140
Training volume (km/week)	63.0	32.1	50.9	2–28
VO_2MAX_ (ml⋅kg^–1^⋅min^–1^)	63.2	6.4	10.2	50.5–74.5
HR_*MAX*_ (bmp)	184.2	10.6	5.7	166–201
VT_1_ (ml⋅kg^–1^⋅min^–1^)	37.6	5.9	15.7	23.1–50.7
VT_2_ (ml⋅kg^–1^⋅min^–1^)	53.5	6.1	11.3	35.9–62.7
RE 12 (ml⋅kg^–1^⋅min^–1^)	41.1	3.1	7.6	37.4–49.1
RE 12 (%VO_2MAX_)	65.1	5.5	8.5	52.8–74.2
RE 16 (ml⋅kg^–1^⋅min^–1^)	52.7	3.3	6.3	46.2–61.5
RE 16 (%VO_2MAX_)	83.5	7.1	8.5	69.4–93.1
C_*MET*_ 12 (J⋅kg^–1^⋅m^–1^)	3.7	0.3	8.7	3.3–4.6
C_*MET*_ 16 (J⋅kg^–1^⋅m^–1^)	3.7	0.2	6.9	3.3–4.4
SJ (cm)	30.3	4.7	15.6	22.6–39.1
PO-SJ (W)	1604	202	12.6	1272–1955
PO-SJr (W⋅kg^–1^)	23.9	1.9	7.9	20.6–27.2
CMJ (cm)	31.6	4.6	14.4	25.2–39.8
PO-CMJ (W)	1634	210	12.9	1298–1964
PO-CMJr (W⋅kg^–1^)	24.4	1.8	7.2	21.8–27.4
EUR-HJ (CMJ-SJ)	1.7	1.4	83.3	−0.8 to 4.4
EUR-PO (CMJ-SJ)	45.9	39.1	85.1	−17.9 to 113.6
EUR-HJ (ΔCMJ-SJ)	5.8	4.6	79.9	−2.2 to 13.1
EUR-PO (ΔCMJ-SJ)	2.8	2.3	80.1	−1.1 to 6.4
EUR-HJ (CMJ/SJ)	1.1	0.1	4.4	1.0–1.1
EUR-PO (CMJ/SJ)	1.0	0.0	2.2	1.0–1.1
vVO_2MAX_ (km⋅h^–1^)	20.6	1.5	7.4	19–25

*VO_2MAX_, maximal oxygen uptake; HR_*MAX*_, maximal heart; VT_2_, second ventilatory threshold; VT_1_, first ventilatory threshold; RE, running economy; C_*MET*_, metabolic cost; SJ, squat jump; PO-SJ, power output of squat jump; PO-SJr, relative power output of squat jump; CMJ: countermovement jump; PO-CMJ: power output of countermovement jump; PO-CMJr, relative power output of countermovement jump; EUR-HJ, eccentric utilization ratio-jump height; EUR-PO, eccentric utilization ratio-power output; vVO_2MAX_, maximum running speed.*

Table 2 shows the contribution of positive VO_2MAX_ and negative RE 16 km⋅h^–1^ (81%) during incremental running test.

**TABLE 2:** Predictors of performance during maximal incremental running test (vVO_2MAX_).

Dependent variable	***r*^2^**	***P***	**Indicator**	**Standardized coefficients (β)**	***p***
vVO_2MAX_ (km⋅h^–1^)	0.81	<0.001	VO_2MAX_	0.95	<0.001
			RE 16 km⋅h^–1^	−0.85	<0.001

*VO_2MAx_, maximal oxygen uptake; RE, running economy.*

**FIGURE 2:**
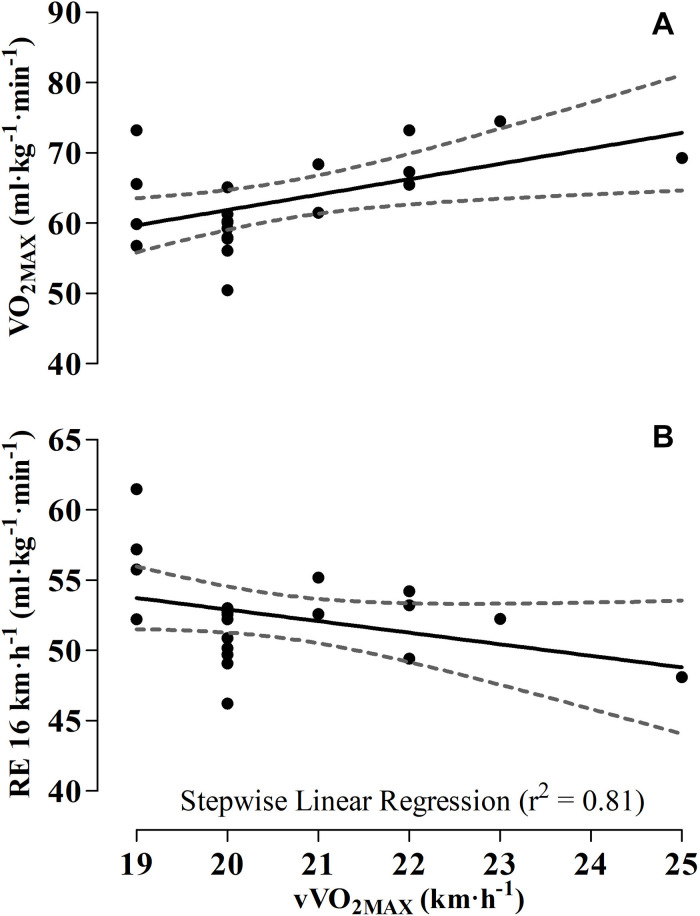
Predicted improvement maximal incremental of running performance (vVO_2MAX_) with VO_2MAX_ **(A)** and RE **(B)**.

Figure 2 shows the contribution of positive VO_2MAX_ and negative RE 16 km⋅h^–1^ (81%) during incremental running test.

## Discussion

The purpose of this study was to investigate the role of physiological parameters on predicting vVO_2MAX_ in recreational runners. The primary finding of this study is that the main determinants of running performance are VO_2MAX_ and RE at 16 km⋅h^–1^. It is worth noting that the RE evaluated at speed close to vVO_2__*MAX*_ (16 km⋅h^–1^) was better related than RE at low speed (12 km⋅h^–1^). Importantly, this study examined, for the first time, the determinants of vVO_2MAX_ in recreational runners, demonstrating that physiological but not neuromuscular factors were associated with performance in this condition.

Indeed, the VO_2MAX_ and RE at 16 km⋅h^–1^ (Table 2 and Figure 2) accounted for 81% of the variance in performance of incremental running test (vVO_2MAX_). Conversely, the C_*MET*_, VT_1_, VT_2_, SJ and CMJ (height and power output) and EUR (height and power output) did not enter in the regression model to predict vVO_2MAX_. These results complement what have already been found in the literature as predictors of maximal endurance running performance. These results agree with previous studies, which indicate VO_2MAX_ and RE, as two of the main predictors of running endurance performance (Conley and Krahenbuhl, 1980; Bassett and Howley, 2000; McLaughlin et al., 2010; Kipp et al., 2019). In general, the relationship between VO_2MAX_ and running performance was similar to other studies using well-trained runners, confirming its importance as a physiological variable linked to distance running performance (Costill et al., 1973; Noakes et al., 1990; McLaughlin et al., 2010). According to McLaughlin et al. (2010), VO_2MAX_ explained 81% of the total variance, and RE accounted for an additional 11% of the 16-km endurance running performance.

The running performance is related to better RE (e.g., a lower VO_2_ for a given running speed) may be worthwhile, especially in endurance events, once it will allow a lower fractional VO_2MAX_ utilization for any submaximal intensity exercise (Daniels, 1985; Barnes and Kilding, 2015). Therefore, runners with the same VO_2MAX_ can present different performances, and this can be explained in part by the RE, as demonstrated here. Results of the current study are in line with previous findings (Noakes et al., 1990; Tartaruga et al., 2012, 2014). For example, Noakes et al. (1990) found a strong correlation between performance and RE (*r* = 0.76–0.90), without regression test to identify the determination percentage between RE and running performance. Therefore, several physiological and biomechanical factors seem to influence RE in highly trained runners and must be taken into account to enable improvements in RE. These factors include metabolic adaptations within the muscle such as increased mitochondria and oxidative enzymes (Saunders et al., 2004), the ability of the muscles to store and release elastic energy by increasing the stiffness and other key parameters of elastic mechanism (da Rosa et al., 2019), more efficient mechanics leading to less energy wasted on braking forces and high vertical oscillation and stride frequency and stride length (Saunders et al., 2004; Tartaruga et al., 2009, 2012).

However, the multiple linear regression test showed no relationship between the C_*MET*_ and the performance in a maximal incremental test. In addition to the expected collinearity between RE and C_*MET*_, the higher regression coefficient between RE and performance than between C_*MET*_ and performance is probably due to the role of basal metabolism contributing to this relationship (Saibene and Minetti, 2003). Moreover, training with vertical jumps promotes strength and power and consequently can improve RE and performance (Kubo et al., 2007; Balsalobre-Fernandez et al., 2016). The results of the present study demonstrate that there is no relationship between the neuromuscular outcomes from vertical jumps (SJ and CMJ) and the maximum endurance running performance in recreational runners that did not performed previously strength and power training programs. Conversely, according to Loturco et al. (2015), the performance of vertical jumps (SJ and CMJ) had a strong correlation (*r* > 0.8) with the sprinting running performance (100 m). Thus, the use of variables from vertical jumps seems to be more suitable for predicting performance in sprints tests, but not for endurance tests. Collectively, the present findings confirm the role of maximal oxygen utilization and metabolic economy on the vVO_2MAX_ (Billat et al., 2000).

## Limitations

The main physiological limitation of the present study was that blood lactate was not evaluated during the maximal incremental running performance, that would allow to identify if the lactate threshold also and would determine endurance performance of the runners (Kipp et al., 2019). Another limitation of the present study was the fact that all evaluations were performed in a single day and, therefore, the last test performed (maximal incremental test) may have suffered interference from the *a priori* tests causing the establishment of processes of muscular fatigue. However, the athletes evaluated were well trained and an interval of 5 min was adopted between each condition, minimizing the effects of muscle fatigue. It is important to highlight that future studies should control for the following variables that were controlled in our study: runners’ age, experience, training volume and competitive level. The control of these variables reinforces the novel approach we used in this study.

## Future Perspectives

Our analysis here focused solely on the oxygen uptake during maximal incremental running and vertical jumps (SJ and CMJ) performance. Therefore, future studies can evaluate the prediction of endurance running performance through the use of other physiological (e.g., skeletal muscle respiration), neuromuscular (e.g., muscle activation and size) and biomechanical (e.g., mechanics work and efficient) variables. Besides that, the decisive step will be an actual sport-setting test, such as during a running race (e.g., 3000 m), to identify which physiological, neuromuscular and biomechanical variables determine endurance running performance.

## Practical Application

Understanding which variables can be predictors of running performance in a laboratory setting may contribute to athletes, coaches and sport scientists when determining physiological behaviors that may lead to the best performance, despite these predictors may not be the same for the outdoor or sports competition conditions, where other variables physiological and biomechanics may play an important role. These findings suggest that interventions that enhance VO_2MAX_ and RE may increase the runners’ vVO_2_ and improve their running performance.

## Conclusion

In summary, according to the outcomes presented in this study, it can be concluded that maximal aerobic performance prediction depends 81% on VO_2MAX_ and RE at 16 km⋅h^–1^. However, we suggested that further studies should be carried out with these and other physiological and biomechanical variables to determine performance in ecological conditions (e.g., athletics track tests) or during endurance running competitions.

## Data Availability Statement

The raw data supporting the conclusions of this article are available in Supplementary Material.

## Ethics Statement

The studies involving human participants were reviewed and approved by Universidade Federal do Rio Grande do Sul (No. 2.437.616). The patients/participants provided their written informed consent to participate in this study.

## Author Contributions

FL was responsible for the conception, acquisition of data, analysis and interpretation of data, drafting of the manuscript and revising it critically for important intellectual content, and final approval of the version to be submitted, and was the corresponding author. EM and GF were responsible for the acquisition of data and analysis, drafting of the manuscript and revising it critically for important intellectual content, and final approval of the version to be submitted. EM was responsible for the data analysis, critical revision of the manuscript, and final approval of the version to be submitted. LP-T was responsible for the data analysis and design of the study, study orientation, supervision, critical revision of the manuscript, and final approval of the version to be submitted. All authors contributed to the article and approved the submitted version.

## Conflict of Interest

The authors declare that the research was conducted in the absence of any commercial or financial relationships that could be construed as a potential conflict of interest.
